# Manipulating Electrocatalysis using Mosaic Catalysts

**DOI:** 10.1002/smsc.202000059

**Published:** 2021-03-27

**Authors:** Yuting Luo, Sum Wai Chiang, Lei Tang, Zhiyuan Zhang, Fengning Yang, Qiangmin Yu, Baofu Ding, Bilu Liu

**Affiliations:** ^1^ Shenzhen Geim Graphene Center (SGC) Tsinghua-Berkeley Shenzhen Institute (TBSI) and Tsinghua Shenzhen International Graduate School (TSIGS) Tsinghua University Shenzhen 518055 P. R. China; ^2^ Tsinghua Shenzhen International Graduate School (TSIGS) Tsinghua University Shenzhen 518055 P. R. China

**Keywords:** catalysts, catalytic activity, electrocatalysis, gas-liquid-solid interface, hydrogen evolution

## Abstract

Understanding the mechanisms and developing strategies toward efficient electrocatalysis at gas–liquid–solid interfaces are important yet challenging. In the past decades, researchers have devoted many efforts to improve catalyst activity by modulating electronic properties of catalysts in terms of chemical components and physical features. Herein, a mosaic catalyst, which is defined as a catalyst with spatially isolated and periodically distributed active areas, is developed to dramatically improve the activity of catalysts. Taking Pt catalyst as an example, the mosaic Pt leads to high catalytic performance, showing a specific activity 11 times higher than that of uniform Pt films for hydrogen evolution reaction (HER), as well as higher current densities than commercial Pt/C and uniform Pt films. Such a strategy is found to be general to other catalysts (e.g., 2D PtS) and other reactions (e.g., oxygen evolution reaction). The improved catalytic performance of the mosaic catalysts is attributed to enhanced mass transferability and local electric field strength, both of which are determined by the occupation ratios of catalysts. The work shines new light on manipulating electrocatalysis from the perspective of the spatial structures of catalysts, which guides the design of efficient catalysts for heterogeneous reactions.

## Graphical abstract

Developing strategies toward efficient electrocatalysis is crucial to chemical production and energy conversion systems. Instead of focusing on the chemical component and nanostructure of catalysts, a mosaic catalyst strategy is developed to improve the activity of electrocatalysts by engineering their spatial structures, which is another degree of freedom to modulate the performance of catalysts.

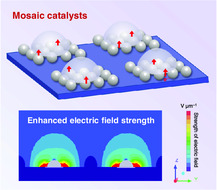

## 1 Introduction

Heterogeneous catalytic reactions play central roles in the production of chemicals and in energy‐conversion systems.^[^ ^1^ ^]^ Much attention has been paid to improve the efficiency of heterogeneous catalysts under electrochemical environments for producing gas fuels, such as hydrogen (H_2_), oxygen (O_2_), methane (CH_4_), and carbon monoxide (CO).^[^ ^2^, ^3^ ^]^ For example, the electrochemical hydrogen evolution is considered attractive due to the cleanliness and high energy density of hydrogen. Note that these heterogeneous catalytic reactions are usually slow in kinetics, making development of high‐efficiency catalysts the key in these fields.^[^ ^4^, ^5^, ^6^ ^]^ In recent years, the community has witnessed many successes in developing efficient electrocatalysts, including Pt and other noble metals, metal oxides, chalcogenides, carbides, and phosphides, among many others, for the gas‐involved electrochemical reactions.^[^ ^7^, ^8^, ^9^, ^10^, ^11^, ^12^, ^13^, ^14^ ^]^ These works have mostly focused on engineering chemical compositions (e.g., doping, alloying, and phase‐changing engineering) and/or physical features (e.g., size, shape, and strain engineering) of the catalysts, by which the bonding strength between reaction intermediates and catalysts can be modulated so as to improve the catalytic performance.^[^ ^15^, ^16^, ^17^, ^18^, ^19^, ^20^, ^21^ ^]^ For example, Lukowski et al. reported that molybdenum disulfide shows a decreased overpotential for hydrogen evolution reaction (HER) when changed from semiconducting 2 H phase to metallic 1 T phase.^[^ ^22^ ^]^ In another work, the electronic structures of atomically dispersed Ru catalysts are precisely modulated by coating metal shells, which exert a proper compressive strain on Ru, resulting in a low overpotential of 220 mV@10 mA cm^−2^ for oxygen evolution reaction (OER).^[^ ^23^ ^]^ These methods are interesting, while modulating the electronic structures of catalysts via chemical compositions and physical features at the atomic level is challenging. It is therefore interesting to explore a simple strategy to engineer the performance of electrocatalysts. In general, electrochemical process is affected by factors including the chemistry, microscopic structure, geometry of catalysts, etc., which thus can be used to improve catalytic performance.^[^ ^5^, ^24^, ^25^, ^26^, ^27^, ^28^, ^29^ ^]^ This fact gives new opportunities in modulating the performance of electrocatalysts besides chemistry.

Here, instead of focusing on the chemical component and nanostructure of the catalysts, we design a type of mosaic catalyst and demonstrate that the spatial structure of the catalyst is another degree of freedom to modulate the performance of electrocatalysts. There are three advantages of engineering the spatial structures of catalysts in improving their performance. First, the design of spatial structures can be combined with other strategies (e.g., doping, alloying, and strain engineering) to further improve the catalytic performance. Second, this method is suitable for engineering catalysts working at high current densities. Third, this method is facile and easy to implement. Taking Pt as an example, we show that mosaic Pt (M‐Pt) exhibits a 11‐times‐higher specific activity for HER than the uniform Pt films and higher current densities than commercial Pt/C and uniform Pt films at the same overpotentials. Such a mosaic catalyst design strategy has further been extended to 2D platinum sulfide (PtS) and Ru catalysts for HER and OER, respectively, showing good universality. Experimental and simulation studies show that the fast mass transfer and enhanced local electric field on these mosaic catalysts endow their improved activity. These results indicate that engineering the spatial structure of catalysts is an effective way for improving the activity of catalysts.

## 2 Results and Discussion

### 2.1 Modulating Spatial Structure of Mosaic Electrocatalyst for Gas‐Involved Reactions

**Figure 1:**
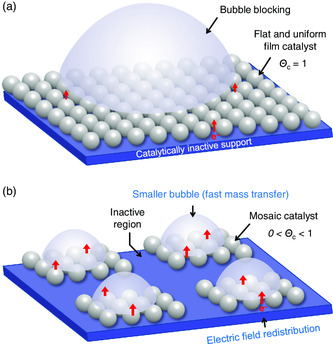
a,b) Engineering spatial structures of electrocatalysts. Schematic showing the adhesion of gas bubbles and the electric field distribution on a) a flat and uniform film electrode, and b) a mosaic catalyst.

The basic principle of modulating the electrocatalysis by the spatial structures of catalysts is shown in **Figure** **1**, where two common factors, mass transfer and the electric field distribution, are considered. First, the adhesion of gas product/reactant bubbles limits the mass transfer rate to catalysts (Figure 1a).^[^ ^20^, ^30^, ^31^, ^32^ ^]^ Catalytic sites may be hindered by these bubbles and become inactive, decreasing the efficiency and performance of catalysts if they are not removed efficiently. Second, a strong local electric field on the catalyst would increase catalytic reaction rate.^[^ ^5^, ^28^, ^33^, ^34^, ^35^, ^36^ ^]^ The electrons taking part in reactions are not only concentrated in the catalyst sites but also in the inactive sites,^[^ ^37^ ^]^ which would decrease the local operating current density and utilization efficiency of the catalyst (Figure 1a).

By modulating the spatial structures of mosaic catalysts, whose catalytic sites and inactive sites are loaded on different regions of the matrix, their mass transfer and the electric field distribution can be redesigned (Figure 1b). The removal efficiency of gas bubbles on the catalyst is mainly controlled by their departure radii (*r*_H_), which determines the excess surface energy (ΔG*) of merged bubbles for detaching the catalyst surface^[^ ^38^ ^]^ and is given by

(1) $$\Delta G^{*}(r_{H})=\sum G_{c}-\sum G_{c}^{\prime }-\sum E_{\text{vis}}$$

where $\sum G_{c}$, $\sum G_{c}^{\prime }$, and $\sum E_{\text{vis}}$ are the total surface free energies of bubbles before and after coalescence and total viscous dissipation energy for each bubble, respectively. A high ΔG* would be obtained when the *r*_H_ of bubbles is small and equal to each other.^[^ ^39^ ^]^ To this end, the geometrical parameters of the catalytic regions (where bubbles nucleate) and the inactive regions are well redesigned (Figure 1b), so as to remove bubbles more efficiently and frequently expose the catalytic sites. The strength of the local electric field on the catalyst can also be redistributed by modulating the spatial structure of catalysts. Electrons are accumulated on the catalyst sites rather than the inactive sites, which improve the catalyst use efficiency (Figure 1b). These effects result in an increase in the thermodynamic driving force for electrochemical reactions and thus an increase of activity on suitably designed catalysts. To identify these mosaic catalysts, the occupation ratio of catalysts (Θ_c_) is proposed and is defined by Θ_c_ = *A*_catalyst_/*A*_electrode_, where *A*_catalyst_ and *A*_electrode_ are the surface areas of catalyst and electrode, respectively. Θ_c_ is equal to 1 for a flat and uniform catalyst (Figure 1a), whereas it is in the range of 0–1 for a mosaic catalyst (Figure 1b).

### 2.2 HER Performance of Pt Electrocatalysts with Spatial Structure Manipulation

**Figure 2:**
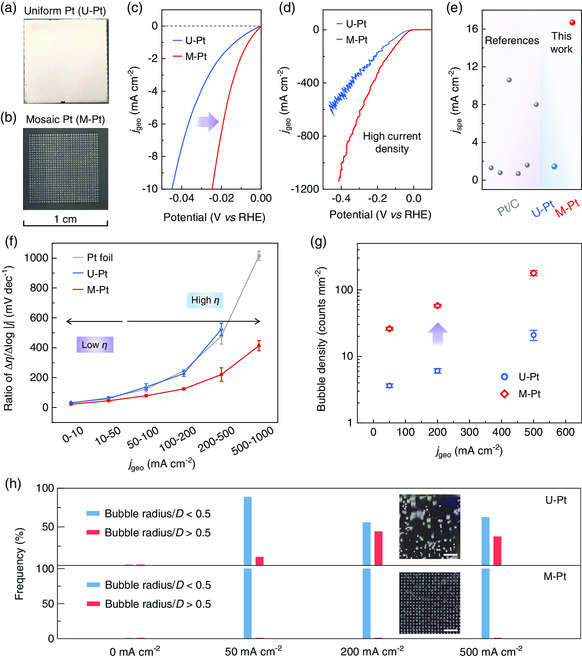
HER performance of different Pt catalysts. a,b) Photos showing (a) a uniform and flat film Pt catalyst (U‐Pt) and b) a mosaic Pt catalyst with its spatial structure well designed (M‐Pt) on glassy carbon electrodes. c) LSV curves of two types of Pt catalysts, showing their geometric current densities (*j*_geo_) and revealing a better intrinsic activity of M‐Pt than U‐Pt. d) LSV curves and *j*_geo_ of Pt catalysts at large overpotentials. e) Specific activities (*j*_spe_) of several Pt samples at an overpotential of 50 mV. f) Values of Δ*η*/Δlog|*j*| (*R*_*η/j*_) for Pt catalysts in different current density ranges. All points are measured three times, and error bars correspond to the standard deviations. g) Densities and h) diameters of H_2_ bubbles on U‐Pt and M‐Pt at different current densities. Here *D* is the distance between adjacent Pt regions in M‐Pt. All points are tested three times, and error bars correspond to standard deviations. Scale bars in the insets of Figure 2 h are 1 mm.

The advantage of the catalyst with a well‐designed spatial structure is tested by Pt catalysts for the HER. Two types of Pt catalysts are fabricated on glassy carbon electrodes, i.e., a uniform and flat Pt film catalyst (U‐Pt, **Figure** **2**a) and a mosaic Pt catalyst with a designed spatial structure (M‐Pt, Figure 2b). The M‐Pt consists of square catalyst regions with edge lengths (*L*) of 125 μm and distances between adjacent regions (*D*) of 250 μm. All Pt catalysts are prepared by the same sputtering deposition method (see “Methods” for details). Atomic force microscopy (AFM) measurements show that the root‐mean‐square (RMS) roughness of the Pt regions on M‐Pt is 1.228 nm, similar to U‐Pt (1.216 nm, Figure S4, Supporting Information). The thickness and morphology of M‐Pt and U‐Pt are kept the same, so that spatial structure is the only difference between the two samples. A Pt foil and a commercial Pt/C film catalyst are also used besides U‐Pt as references.

We test the HER performance of these catalysts in a 0.5 m H_2_SO_4_ solution. Typical linear sweep voltammetry (LSV) curves reveal that M‐Pt has a high catalytic performance for HER. Specifically, their intrinsic activities are first compared where current densities are determined by geometrical surface areas of Pt (*j*_geo_).^[^ ^40^, ^41^ ^]^ All the samples show a Tafel slope close to 30 mV dec^−1^, indicating that they follow the same rate‐determining recombination step (Figure S5, Supporting Information). Interestingly, M‐Pt has a smaller overpotential (*η*) than the others, reduced by 21 mV (47%) in overpotential than U‐Pt at a current density of 10 mA cm^−2^ (Figure 2c). These results show that M‐Pt has a higher catalytic activity than U‐Pt though they have identical chemical compositions. At large overpotentials, M‐Pt shows a much higher *j*_geo_ than other samples, with a *j*_geo_ of 1000 mA cm^−2^ at ≈400 mV, which is about twice of the U‐Pt (Figure 2d). Then, we test the electrochemically active surface areas (ECSAs) of these Pt samples using their measured charge of hydrogen desorption peaks after double‐layer correction. The specific activities (*j*_spe_) of the samples that are determined by respective ECSAs are compared (Figure 2e). The results show that *j*_spe_ of M‐Pt is 16.7 mA cm^−2^ at *η* = 50 mV, which is compared favorably with the Pt foils and commercial 20 wt% Pt/C catalysts (Figure 2e and Table S2, Supporting Information) and is about one order of magnitude higher than U‐Pt.

We further analyze the catalytic performance of the Pt catalysts at different current densities. The value of Δ*η*/Δlog|*j*| (*R*_*η/j*_, defined as overpotential *η* divided by current density *j*), a recently proposed indicator to evaluate the performance of catalysts at different current densities,^[^ ^20^ ^]^ is used to evaluate how much overpotential is needed as the current density increases (Figure 2f). *R*_*η/j*_ of M‐Pt remains small (414 mV dec^−1^) but becomes large for U‐Pt and Pt foil (1015 mV dec^−1^) as the current density increases to 1000 mA cm^−2^, indicating that M‐Pt maintains its performance at high current densities. We also compare the mass transfer on U‐Pt and M‐Pt at different current densities (Figure 2g). Although the densities of H_2_ bubbles on both catalysts increase with the current density, M‐Pt shows two orders of magnitude greater bubble density than that of U‐Pt. At a small current density of 10 mA cm^−2^, the majority of H_2_ bubbles on both samples have diameters smaller than 250 μm, which is close to *D* (distance between adjacent Pt regions in M‐Pt catalyst). As current density increases, more active sites are blocked by the grown bubbles on U‐Pt, whereas the sizes of bubbles on M‐Pt remain less than *D* due to high bubble removal efficiency (Figure 2h). In situ optical microscopy (OM) observations show that bubbles on M‐Pt are removed at a high rate in a “bubble‐relay” mode, whereas bubbles on U‐Pt are randomly generated and removed in a slow rate (Movie S1, Supporting Information). According to formula (1), the higher density and smaller size of hydrogen bubbles (i.e., smaller *r*_H_) on M‐Pt than on U‐Pt promote the mass transfer efficiency of H_2_ on M‐Pt. Overall, these results indicate that M‐Pt shows an improved HER performance.

### 2.3 Universality of Catalyst Design toward Improving Catalytic Performance

**Figure 3:**
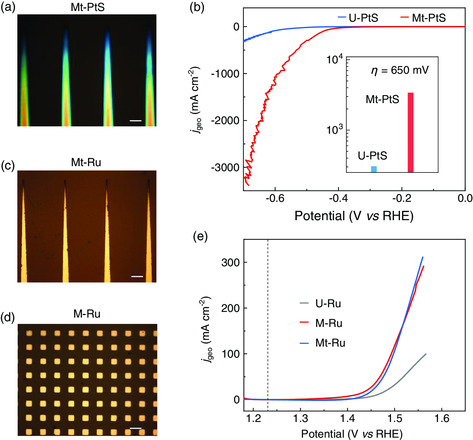
Universality of the catalyst design strategy for different catalysts and reactions. a) An OM image of Mt‐PtS catalyst. b) LSV curves of U‐PtS and Mt‐PtS for HER in 0.5 m H_2_SO_4_. c,d) OM images of c) Mt‐Ru and d) M‐Ru catalysts. e) LSV curves of U‐Ru, M‐Ru, and Mt‐Ru catalysts for OER in 0.5 m H_2_SO_4_. All scale bars are 200 μm.

As a demonstration of the universality of such a catalyst design strategy, we extend it to other catalysts and gas‐involved reactions. The first example is PtS for the HER. The PtS is grown by chemical vapor deposition (CVD) into either a uniform film catalyst (U‐PtS) or mosaic catalyst with the redesigned triangle‐shaped patterns (Mt‐PtS, **Figure** **3**a and S6, Supporting Information, see “Method” for details). We find that Mt‐PtS shows a much better HER performance than U‐PtS. For example, Mt‐PtS shows a *j*_geo_ = 3329 mA cm^−2^ at *η* = 650 mV (Figure 3b), whereas U‐PtS only delivers 305 mA cm^−2^.

The second example is Ru catalyst for the OER. Three Ru catalysts are prepared, including two mosaic catalysts with either triangle or square patterns (M‐Ru and Mt‐Ru) and one uniform and flat film catalyst (U‐Ru, Figure 3c, d, see “Methods” for details). The polarization curves show that M‐Ru and Mt‐Ru have better OER performance than U‐Ru (Figure 3e). For example, Mt‐Ru shows a *j*_geo_ = 183 mA cm^−2^ at *η* = 320 mV, which is three times higher than that of U‐Ru at the same overpotential. These two examples show that the catalyst design strategy is general to other materials (PtS) and other reactions (OER), besides Pt for HER.

### 2.4 Tuning Catalytic Performance of Catalysts by Engineering Their Spatial Structures

**Figure 4:**
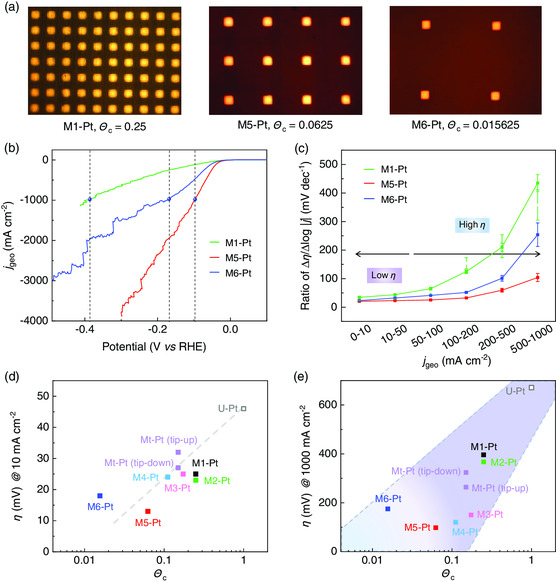
Tuning HER performance of mosaic Pt catalysts by engineering their spatial structures. a) OM images showing three Pt catalysts with different Θ_c_, marked as M1‐Pt (Θ_c_ = 0.25), M5‐Pt (Θ_c_ = 0.0625), and M6‐Pt (Θ_c_ = 0.015625). b) LSV curves of the three representative Pt catalysts. c) *R*_*η/j*_ for the three Pt catalysts at different current density ranges. All points were tested three times, and the error bars correspond to the standard deviations. d,e) Summaries of d) *η*_10_ and e) *η*_1000_ values of Pt catalysts with different Θ_c_ values, where *η*_10_ and *η*_1000_ are defined as overpotentials at *j*_geo_ = 10 mA cm^−2^ and *j*_geo_ = 1000 mA cm^−2^, respectively.

We then show how the geometrical features of the catalyst affect their performance. Six mosaic Pt catalysts (namely, M1‐Pt, M2‐Pt, M3‐Pt, M4‐Pt, M5‐Pt, and M6‐Pt) with sequentially decreased Θ_c_ are fabricated (**Figure** **4**a and Table S2, Supporting Information) for HER tests. From their LSV curves, all the M‐Pt catalysts show better HER performance than Pt foil and U‐Pt. Among them, M5‐Pt shows the best performance with *η* = 13 mV at *j*_geo_ = 10 mA cm^−2^ and *η* = 98 mV at *j*_geo_ = 1000 mA cm^−2^ (Figure 4b). In contrast, M1‐Pt, M6‐Pt, and Pt foil exhibit large overpotentials of 25, 18, and 46 mV at *j*_geo_ = 10 mA cm^−2^ as well as 396, 175, and 671 mV at *j*_geo_ = 1000 mA cm^−2^, respectively (Figure S5 and S7, Supporting Information). We further analyze *R*_*η*/*j*_ ratios of these Pt catalysts (Figure 4c). With increasing current density, M1‐Pt and M6‐Pt have large *R*_*η/j*_ ratios of more than 200 mV dec^−1^ whereas M5‐Pt has a small *R*_*η*/*j*_ ratio of less than 100 mV dec^−1^. All these results indicate that engineering the spatial structures of catalysts is an easy and effective way to improve heterogeneous catalytic performance.

The parameter Θ_c_ is used to describe the spatial structure of the catalysts. By summarizing the overpotentials at both low (10 mA cm^−2^, i.e., *η*_10_) and high *j*_geo_ (1000 mA cm^−2^, i.e., *η*_1000_) of samples and their different Θ_c_ values, we study how factors influence their catalytic performance, as shown in Figure 4d‐e and S8, Supporting Information. The results indicate that both *η*_10_ and *η*_1000_ show the correlations with Θ_c_ values (Figure 4d‐e). At smaller Θ_c_ values, *η*_10_ reduces and that means a better catalytic performance. Moreover, the correlation between overpotential and Θ_c_ value is not a simple one. In the case of 1000 mA cm^−2^, the approximately linear correlation between overpotential and Θ_c_ value is not as clear as low current density (Figure 4e). Such a deviation from the correlation can be attributed to great contribution of mass transfer at high current densities. Relationships between other structural parameters of Pt catalysts and their catalytic performances are also studied. The results show no obvious correlations between catalytic activity and wettability of electrodes (Figure S9, Supporting Information), as well as the ratio of catalyst perimeter to area, meaning the edges of Pt regions contribute negligibly to the HER activity of M‐Pt (Figure S10, Supporting Information). The above results indicate that a properly low Θ_c_ increases the efficiency of each catalyst region and improves the performance of electrocatalysts.

### 2.5 Effect of Size of H_2_ Bubbles on Mass Transfer and HER Performance

**Figure 5:**
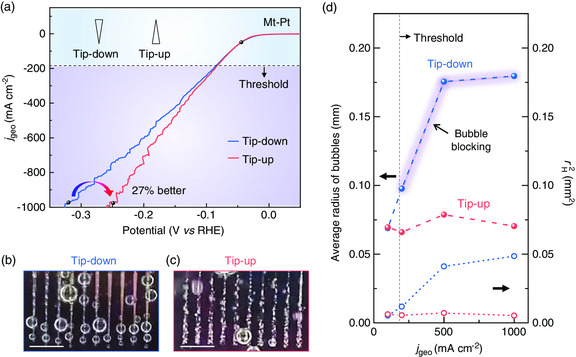
Effect of bubble size on mass transfer and HER performance. a) LSV curves of Mt‐Pt with tip‐up (red) and tip‐down (blue) orientations. b,c) Photos showing different transfer abilities of H_2_ bubbles on b) tip‐down and c) tip‐up samples. By changing catalyst orientations, their performance changes due to different mass transfer abilities. Scale bars in (b) and (c) are 2 mm. d) Relationships between average radius (left *Y*‐axis) and $r_{H}^{2}$ (right *Y*‐axis) of H_2_ bubbles to HER performance, indicating a critical bubble radius of ≈100 μm, over which H_2_ bubbles impede the mass transfer process in Mt‐Pt samples.

Having verified the effectiveness of modulating the spatial structure of catalysts to improve their activity, we now explore the contribution of bubble size to mass transfer and catalytic performance. Two opposite‐placed orientations of triangle‐shaped Pt catalysts (Mt‐Pt) are used with the tips of triangle regions up or down, referring to the direction of gas flow, i.e., tip‐up and tip‐down (**Figure** **5**a and Figure S11, Supporting Information). The two samples show the same chemical composition and morphology, making orientation as the only different parameter. As a result, the two samples show the opposite directions of the Laplace forces on the bubble caused by the asymmetrical triangle shapes of the catalyst regions (Figure S12, Supporting Information),^[^ ^42^ ^]^ and thus the effect of mass transfer on catalytic performance can be independently studied. As shown in Figure 5a, both samples show the same catalytic performance at current densities <200 mA cm^−2^, whereas the tip‐up sample shows better performance than the tip‐down one at current densities >200 mA cm^−2^ (Movie S2, Supporting Information). As current densities get higher, their HER difference becomes more significant. For example, the overpotential at *j*_geo_ = 1000 mA cm^−2^ decreases by ≈27% for tip‐up Mt‐Pt compared with tip‐down Mt‐Pt. We also verify the role of mass transfer on high‐current‐density HER by intentionally blocking the removal of H_2_ bubbles with a piece of aerophilic polytetrafluoroethylene on M‐Pt catalyst (Figure S13, Supporting Information). These results show that the mass transfer rate plays a key role in determining the catalytic performance of catalysts at high current densities.

The square of departure radii of bubbles, $r_{H}^{2}$, is related to blocked areas of the catalysts following the Cassie–Baxter mode (see “Methods” for details), which is used to evaluate the critical radius of H_2_ bubbles that influenced mass transfer ability (Figure 5b‐c and S11, Movie S2, Supporting Information). The radii of bubbles are obtained by analyzing tens of H_2_ bubbles (Figure S14, Supporting Information). The results show that $r_{H}^{2}$ of H_2_ bubbles on tip‐down Mt‐Pt is larger than that on the tip‐up one (Figure 5d). As shown in Figure 5a, the current density at critical point is about *j*_geo_ = 200 mA cm^−2^, referring to a critical $r_{H}^{2}$ of ≈10^4^ μm^2^ and an average bubble radius of 100 μm. Oversized H_2_ bubbles will impede mass transfer on the catalyst and increase the mass transfer overpotential of the whole reaction system, resulting in low catalytic performance.^[^ ^40^ ^]^ Taken together, these results show the importance of mass transfer on catalytic performance, especially at high current densities.

### 2.6 Electric Field Redistribution

**Figure 6:**
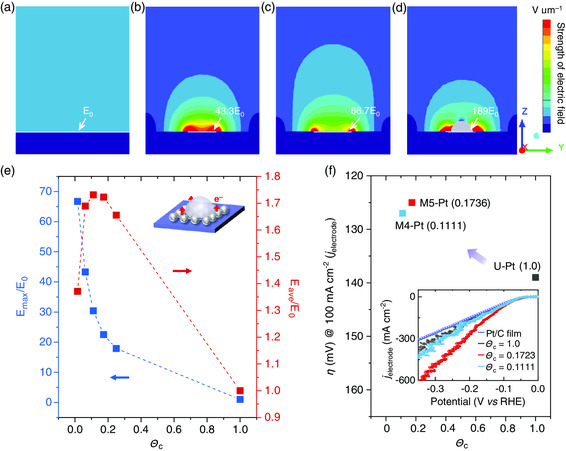
Theoretical analysis. a–d) Local electric field distribution in electrolyte in different catalysts, including a) U‐Pt, b) M‐Pt with Θ_c_ = 0.0625, c) M‐Pt with Θ_c_ = 0.1111, and d) a hemispheric gray bubble with a diameter of 100 μm at the center of M‐Pt with Θ_c_ = 0.0625. The strength of the electric field on U‐Pt (denoted as light blue color) is *E*_0_ and set as the reference. The Pt regions are shown by white lines. e) The correlations between Θ_c_ of M‐Pt and their *E*_max_/*E*_0_ and *E*_ave_/*E*_0_, where *E*_max_ and *E*_ave_ are the maximum electric field and average electric field based on electrode surface areas. f) M‐Pt samples show overpotentials comparable with those of U‐Pt and commercial 20 wt% Pt/C film catalyst at an electrode current density (*j*_electrode_) of 100 mA cm^−2^. The inset shows their polarization curves.

Besides mass transfer, we study the electric field redistributions of different M‐Pt electrocatalysts by the finite element analysis (FEA). As a reference, U‐Pt is also studied. The method and theory used in the analysis are discussed in “Simulations of the Electric Field Distribution” in the Supporting Information. First, local electric field distribution is explored. The results show that electric field is uniformly distributed on U‐Pt with a strength of *E*_0_ (**Figure** **6**a). Here, *E*_0_ denotes the strength of electric field (E‐field strength) on a U‐Pt system. As for M‐Pt, the Pt regions can act as a “point sink” of the electrical potential on the catalytically inactive carbon, which does not participate in the electric current flows. According to potential theory, this small sink‐like structure can greatly enhance local E‐field strength close to these Pt regions (Figure 6b) (see Supporting Information for details). As a result, the electrical energy transport congests into the electrode through these small M‐Pt regions and causes the electric field strengthening localized at their vicinity. This enhancement would result in reaction improvement on M‐Pt by providing more electrical energy to the reactions from the enhanced electric field.^[^ ^5^, ^33^, ^36^, ^43^ ^]^ Moreover, the strength of the electric field is sensitive to the coverage and exposure of M‐Pt (Figure 6c). For the three‐phase‐interface‐involved reactions such as HER, we also study the effect of gas bubbles on the distribution of electric field and the results indicate that the strength of local electric field can be further enhanced by the existence of gas bubbles (Figure 6d and Figure S15, Supporting Information). A similar enhancement by these small bubbles is also predicted in FEA simulations. Note that the effect of mass transfer is not considered in the simulation as the dynamic process is usually complicated. In addition to the local electric field distribution, we have also considered the maximum achieved electric field strength, *E*_max_, near the Pt regions in different M‐Pt and U‐Pt. A normalized representation of *E*_max_/*E*_0_ is used as an indicator to describe how strong the maximum electric field is in each reaction system.^[^ ^34^ ^]^ We plot the values of *E*_max_/*E*_0_ of different M‐Pt with their corresponding Θ_c_ (i.e., *L* ^2^/*D* ^2^) for the square‐shaped catalysts (see Supporting Information for details). The results show that the magnitude of *E*_max_/*E*_0_ of M‐Pt can reach several tens of number of times of enhancement (Figure 6e). In theory, the increase in *E*_max_ will improve local reaction significantly due to the catalytic reaction enhancement from stronger electric field.^[^ ^28^, ^40^ ^]^ (see Supporting Information for discussions). The simulation analyses provide quantitative results and locations of E‐field enhancement by small Θ_c_. These simulation results support our experimental observation of great local reaction enhancement near the M‐Pt regions.

Furthermore, the average values of electric field strength over the electrode area (including areas of Pt regions and areas of inactive regions, *E*_ave_) are analyzed to study geometric activities of catalysts based on the electrode areas. We find that *E*_ave_/*E*_0_ reaches the peak when Θ_c_ is in range from 0.1 to 0.3 (Figure 6e). The enhancement of the reactions therefore should not be linear and M‐Pt with a proper occupation of the catalyst may have a high electrode activity for reactions, giving opportunities to future engineering of spatial structures of catalysts. Note that the optimized ranges for obtaining the best electrode activity will change by the geometrical parameters and microscopic morphologies of each catalyst region. Here, the electrode current density determined by the project area of the electrode (*j*_electrode_) is used for comparison as it is an index for practical use. Guided by this feature, we find that optimized M‐Pt samples (with Θ_c_ of 0.1736 or 0.1111) show overpotentials much smaller than those of U‐Pt and commercial Pt/C film catalyst at *j*_electrode_ = 100 mA cm^−2^ (Figure 6f and S16, Supporting Information). This design strategy is particularly meaningful for expensive catalysts. Together, our simulation results show that the improved catalytic activity of M‐Pt is ascribed to the strengthening of local electric field.

## 3 Conclusion

We have demonstrated a mosaic catalyst for efficient electrocatalysis, based on a new degree of freedom of catalyst design, i.e., their spatial structures. Two key considerations in such a catalyst design are the local electric field strength and mass transfer ability, both affected by the spatial structures of the catalyst. Besides the HER and OER reported here, this catalyst design may have universal relevance to various catalytic reactions involving gas‐involved heterogeneous reactions, including carbon dioxide electrochemical reduction, nitrogen electrochemical reduction, and oxygen reduction reaction, because mass transfer rate and electric field redistribution are common features in these processes.

## Conflict of Interest

B.L., Y.L., and Z.Z. declare that the patents related to this research have been filed by Tsinghua‐Berkeley Shenzhen Institute, Tsinghua University. The University's policy is to share financial rewards from the exploitation of patents with the inventors.

## Data Availability Statement

Research data are not shared.

## Supporting information

Supplementary Material

Supplementary Material

Supplementary Material
