# Supplementary material for: Manipulating Electrocatalysis using Mosaic Catalysts
Source: Small Sci. 2021 Mar 27;1(5):2000059. doi: 10.1002/smsc.202000059 (PMC11935826; doi:10.1002/smsc.202000059)
Supplement: Supplementary file 1 — Supplementary Material [file SMSC-1-2000059-s002.pdf]

Supporting Information for

Manipulating Electrocatalysis using Mosaic Catalysts

Yuting Luo[1], Sum Wai Chiang[2], Lei Tang[1], Zhiyuan Zhang[1], Fengning Yang[1], Qiangmin Yu[1], Baofu Ding[1] & Bilu Liu[1,*]

[1] Shenzhen Geim Graphene Center (SGC), Tsinghua-Berkeley Shenzhen Institute (TBSI) & Tsinghua Shenzhen International Graduate School (TSIGS), Tsinghua University, Shenzhen 518055, P. R. China.

[2] Tsinghua Shenzhen International Graduate School (TSIGS), Tsinghua University, Shenzhen 518055, P. R. China.

Correspondence should be addressed to B.L. (bilu.liu@sz.tsinghua.edu.cn)

Content

1. Methods **3**

2. Simulations of the electric field distribution **6**

3. Materials and samples characterization **15**

4. Electrochemical tests **18**

5. Transferability of hydrogen bubbles and the effects **22**

6. Catalytic performance normalized by electrode surface areas **27**

7. Supporting references **28**

1. Methods

Catalyst preparation: Pt or Ru metals were deposited onto a support like glassy carbon using sputtering deposition techniques, including ion sputtering using an auto fine coater (JEOL, JFC-3000FC, Japan) or e-beam evaporation (Shenzhen Tenstar Vacuum Co., Ltd., TSV-550, China). For the mosaic catalysts, different shadow masks were used during the deposition process. U-Pt or U-Ru were fabricated without any masks. M1-Pt, M3-Pt, M4-Pt, M5-Pt, M6-Pt and M1-Ru were fabricated by square patterned shadow masks with different edge lengths (L, edge length of catalyst islands) and distances (D, distance from the center of one square to the center of the nearest square). M2-Pt with small features was fabricated using a direct laser writing system (miDALIX, DaLI, Germany) to generate small L and D values. Mt-Pt and Mt-Ru were fabricated by a triangle-patterned shadow mask. The thicknesses of all the mosaic catalysts and film catalysts was ~20 nm, which was monitored by sensors during film deposition and measured by ellipsometer. U-PtS and Mt-PtS catalysts were prepared by sulfurizing the corresponding Pt samples in a home-made vertical CVD system under H_2S and Ar with flow rates of 30 standard cubic centimeter (sccm) and 500 sccm at 750 °C for 30 min.[1] The detailed geometrical parameters of all catalysts are shown in Table S2. The Pt/C film electrode was prepared by dispersing 20 wt% Pt/C powder (4 mg) in a water/ethanol/Nafion solution (32/7/1 vol, 1 mL), followed by dropping a certain amount of the dispersion onto a glassy carbon electrode with a catalyst loading amount of 1 mg cm^{-2}.

Material characterization: The morphologies of catalysts were examined by optical microscope (Carl Zeiss Microscopy, Germany) and AFM (Cypher ES, Asylum Research, Oxford Instrument, USA). The Raman spectra of PtS_2 were collected using 532 nm laser excitation with a beam size of ~1 μm (Horiba LabRAB HR Evolution, Japan). The contact angles of droplets on the samples were recorded

by a contact angle measuring device (MDTC-EQ-M07-01, Japan). The droplet volume was the same in all cases (4 μL).

Electrochemical measurements: A standard three-electrode electrolyzer with a H_2SO_4 solution (0.5 M) was used in all tests, with a saturated calomel electrode (SCE) connected by a Luggin capillary and a graphite rod as the reference and counter electrodes, respectively. Pt counter electrode was used for taking videos. For fair comparisons, all the samples were tested in a same electrolyzer with identical test parameters, including the size and position of electrodes, the electrochemical parameters, and the same *iR* compensation (85%) for all the samples. Before each test, the electrolyte was bubbled with Ar or O_2. The scan rate was 10 mV s^{-1} for the linear sweep voltammetry (LSV) tests and the scan rate for the cyclic voltammetry (CV) tests was 20 mV s^{-1}. All the LSV data were collected after few cycles and the performance was stable. The electrochemically active surface areas (ECSAs) of these samples were obtained by measuring their charge of hydrogen desorption peaks after the double layer correction (Q_H). The coefficient 210 μC cm^{-2} was used for adsorption charge of monolayer hydrogen on Pt. The ECSAs were calculated as follows,

$$\text{Pt ECSA} = Q_H / (210\ \mu C\ cm^{-2}) \qquad \text{(Equation S1)}$$

Then, the ECSA of each sample was used to obtain their specific activity (j_{spe}). The geometrical current density (j_{geo}) of sample was got via normalizing measured current by each geometrical surface area of Pt catalyst. Meanwhile, their electrode current density ($j_{electrode}$) was obtained via normalizing the measured current by the projected surface area of electrode, as a performance index for practical use.

In situ optical microscope observations: A home-made electrolyzer was used when taking the in-situ optical microscopy movies by using an Ag/AgCl and a Pt wire as the reference and counter

electrodes, respectively. The effect of Pt counter electrodes can be negligible because the test time is short, and the electrolytes were replaced for each run of test. The Pt catalysts were used as the working electrodes. A X5 or X20 lens were used for the observations. The data collected in this system were used for analyzing the radii and density of H_2 bubbles.

Calculations of coverage of H_2 bubbles on catalysts: The coverage areas of H_2 bubbles on the electrocatalysts in Figure S10 were calculated as follows.

$$A_{bubble}/A_{catalyst} = \frac{Contact\ area\ of\ H_2\ bubbles\ on\ catalysts}{Total\ area\ of\ catalysts} = \frac{\sum \pi[Radii\ of\ bubbles \times \sin(180^{\circ} - \theta)]^2}{Total\ area\ of\ catalysts}$$

(Equation S2)

where the radii of H_2 bubbles are the projected radii of the bubbles and θ is their contact angle. The average contact angle of H_2 bubbles on M1-Pt was measured to be ~140°.

2. Simulations of the electric field distribution

Simulation tools.

As shown in previous studies,[2-4] the electric field distribution in an electrochemical system is important in determining the catalytic reaction rate. It is found that a strong local electric field can increase the reaction rate, as shown by previous experimental and simulation results. To avoid confusion with these references, we follow their convention and use "E" to represent the strength of electric field (E-field strength). It is commonly known that a stronger local E-field contributes to reaction rates, as predicted by the first-principle calculations[4] and the electrochemical theory[5] based on fundamentals in classic book written by John Newman[6]. These enable some studies[2, 3, 7] to use finite element analysis (FEA) tools to study electrochemical reaction performance. Our studies also follow the similar approach and employ this theoretical relation between E-field strength and reaction rate under assumption of existence of abundant concentration of reactants. Then, we use FEA tools to simulate E-field distribution in an electrochemical system, followed by comparing the corresponding reaction rates at different locations near the U-Pt and M-Pt electrodes. Briefly, the highly reactive regions that found in experiments are as predicted by the simulations.

To simulate the E-field distribution, we employ the electromagnetic packages in ANSYS. Using the E-field simulation results, the effects of different geometrical factors on reaction rate can be assessed quantitatively. In particular, we observe the reaction enhancement by the Pt region sizes, distance between Pt regions, and the point-sink effects. Effects of bubbles as-generated during some reactions on E-field distribution are also discussed. As follows, we will show our modeling and simulation details of the U-Pt and M-Pt electrode systems, and then show the visualization of results with detailed discussions.

Simulation modeling details.

The simulation models are built to emulate the U-Pt and M-Pt experimental geometries, and the simulation systems are composed of electrodes, electrolytes, and in some cases the bubbles over M-Pt. These models are constructed for observation of the E-field strength distributions surrounding the Pt electrodes and enable us to understand the reaction rate near the M-Pt regions.

One modeling detail concerns the interface between glassy carbon and electrolyte. This interface carries no electrochemical reaction because there is no reduction/oxidation of reactant ions taking place. Therefore, no faradaic current flows through this interface in the normal direction. This means that, the changes of electric potential and E-field strength in the electrolytes are only due to the current flows within electrolyte, driven through by the upper electrode and the lower U-Pt or M-Pt electrodes. By looking at the geometrical parameters shown in Figure S1, it resembles a source-sink scenario with point-sink current outlets, which is a kind of E-field distribution that similar solution is available in classical potential theory[8, 9].

Consider the E-field distribution around a point sink, which forms E-field with a far-away planar source. Due to the similarity in geometry, this E-field solution can be an approximation for our M-Pt system, especially when the M-Pt is relatively small in size. In classical electric potential theory[8, 9], closed form analytic solutions for very simple configuration like this exists, as depicted in (see Equation S3). But for our complex system geometry, such E-field solution requires computer simulation to find. In fact, even for simple geometry as a spherical bubble[8], we do not have closed form analytical solution. So we have to consider the solution in components.

For our geometry, this point-sink solution is the main feature. Under our model assumptions, the bubble, M-Pt shape, and the glassy carbon plane will modify this solution, due to the linear solution property of the Poisson equation. Although the detailed E-field solutions require computational

simulations, fortunately this theoretical point-sink solution can guide us to understand the physics as an approximated solution for our case. This approximate E-field solution suggests us to consider the parameter $\Theta_c = L^2/D^2$, which is related to the areal ratio of Pt regions on the M-Pt-electrode. When it is small, *i.e.,* $\Theta_c \ll 1$, our solution will approach the point-sink potential theory solution.

Consider a small M-Pt patch (*i.e.*, a Pt region on M-Pt) seen from far-field under a macroscopic dimension *D*, the potential and E-field distribution is similar to that of a point-sink when the size *L* of this M-Pt patch is small enough relative to *D*, *i.e.*, $\sqrt{\Theta_c} = L/D$ <<1. The surrounding E-field strength thus behaves like a point sink solution[9],

$$\mathrm{E} \propto \frac{\mathrm{E}_0}{4\pi} \frac{\mathrm{r}}{||\mathrm{r}||^3} \qquad \text{(Equation S3)}$$

Here, $\mathbf{r}$ is the coordinate vector measured from the sink point and E_0 is a reference field strength on U-Pt. This solution carries the essential E-field distribution property that we want to understand. For simplicity, we ignore the other components from the bubble and the lower glassy carbon electrode.

The solution form of the Equation S3 suggests, we see that the value of $\mathbf{r}$ is small near the small M-Pt patch, and we notice that the value of E-field strength at the neighborhood of the M-Pt patch receives large amplification due to the $\mathbf{r}^3$ term at the denominator. In our experiment and simulation, we typically employ the order of value $L/D = 125\ \mu\mathrm{m}/500\ \mu\mathrm{m} = 0.25$, which is sufficiently small to affect a good magnification of E-field surrounding the M-Pt patch.

The solution also informs us of a normalized representation by reference E_0. In the simulation results, we expressed the E-field strength maximum ($\mathrm{E}^*_{\mathrm{max}} = \mathrm{E}_{\mathrm{max}}/\mathrm{E}_0$) and the average E-field ($\mathrm{E}^*_{\mathrm{ave}} = \mathrm{E}_{\mathrm{ave}}/\mathrm{E}_0$) in their corresponding normalized forms by E_0. Here, we choose E_0 to denote the reference uniform E-field strength from the reference case, *i.e.* two flat-plate electrodes (U-Pt or flat Pt film) sandwiching the simulation domain of the same dimension and electrolyte. It is noted that the

E_0 in our experiment is in the range of 0.500 – 0.833 kV/m. The potential settings (V_0) is in the range of 1.5 V – 2.5 V, where we have followed the settings in simulation. Given the same geometry, the scaling of E_0 value will not affect the normalized E-field solutions. As can be seen from Equation S1, the normalized solution does not depend on the value of E_0, which is a frequent property in analytical solutions from potential theory. Therefore, we consider our computational solutions expressed in the normalized form as an appropriate representation.

Computational setup.

For computational efficiency, our models consider a unit-cell region near the U-Pt and M-Pt electrodes, where the main E-field distribution phenomena is observed. The model schematics can be seen in Figure S1. As shown in Figure S1(a), two conductive electrodes (a carbon anode at the top is parallel to a M-Pt or U-Pt cathode at the bottom) sandwiching an electrolyte layer (an aqueous H_2SO_4 solution with sufficient protons) constitute the modeling domain. For cases where Pt metal are plated fully (U-Pt) or for cases only on a square patch (M-Pt) at the bottom electrode, we setup the boundary accordingly. The main boundary conditions are: a potential V_0 is applied between the top electrode and the bottom electrode, and an electric current normal to the Pt patches are allowed.

In case of Figure S1 (b), a hydrogen bubble of designated size and location is situated within the domain, so that its effect on E-field can be observed and compared. In these simulations, the domain size is 1000 μm × 500 μm × 500 μm, which is 1 periodic unit cell as in the experiments. Depending on the interested scenario, the hydrogen bubble is derived from portion of a sphere. For simulation with bubbles, the hydrogen bubble is situated on or near the bottom electrode, often near the Pt-coated patches, which can be U-Pt or M-Pt. We observe and compare the E-fields after applying the potential

difference stated above. The material properties we used are from standard material database measured at standard room temperature environment.

The bubbles size D_{bubble} and their locations shows different blockage effects on the electricity and alter the current flow pathway in the electrolyte. This modifies our primary solution in Equation S3 and a new E-field distribution can be observed. We shall discuss the E-field distribution in the result section. Various cases are simulated, and some selected cases are listed in Table S1.

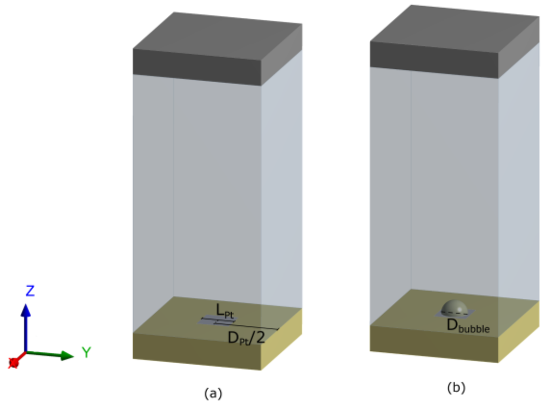

Figure S1. (a) U-Pt or M-Pt cell without a bubble (model a). (b) U-Pt or M-Pt cell with a bubble (model b). L_{Pt} is the size of M-Pt, D_{Pt} is the patch separation, and D_{bubble} is the bubble size. The top and bottom electrodes are shown, and the M-Pt/U-Pt and top electrode in contact with the electrolyte are applied with electricity.

Table S1. Selected simulation cases, with a bubble size and patch size indicated.

Sample No.	Cell type	Patch type	Bubble	Remarks
1	Single cell	U-Pt	No	Model (a)
2	Single cell	M-Pt (various size)	No	Model (a)
3	Single cell	U-Pt	Size = 100 μm	Model (b)
4	Single cell	M-Pt (D = 125 μm)	Size = 100 μm	Model (b)

Results of Case 1. E-field redistribution of U-Pt vs M-Pt in a single cell, without bubble.

The first case is to observe the point-sink E-field enhancement effect from the M-Pt patch using model (a). The effect of parameter Θ_c on the E-field strength is observed and discussed.

First, we discuss the local E-field redistribution. Figure S2 shows the E-field distribution on the Z-Y plane (the plane passing through the centers of the electrodes) in the system. The U-Pt case (Figure S2a) has a very simple feature and shows a very uniform light blue color E-field strength, which is the reference E_0 value. This is considered as a control case, where the system is very simple with two electrodes sandwiching electrolytes. For the small M-Pt patch cases (Figures S2b and S2c), the patch acts like a point-sink of electrical potential when observed from afar. When the distance to the patch (*i.e.*, **r**) is smaller, the E-field becomes stronger when getting nearer to the patch. At the vicinity of the patch, the electrical energy is congesting into the bottom electrode through the small M-Pt patch, which cause this E-field strengthening there. This probably creates the reaction enhancement near the M-Pt plating by providing more electrical energy to the reaction there, as reviewed by F. Che *et al.*[4].

In addition to the local E-field distribution, we have also considered the E_{max} (the maximum observed E-field) and the E_{ave} (the areal average of the E-field over the bottom electrode) near the M-Pt. The E_{max} result can show how the E-field enhanced under different geometrical parameters, and E_{ave} result can present whether the E-field per electrode area has ever been improved. We plot the E_{max}/E_0 and E_{ave}/E_0 vs the Θ_c parameter on Figure 6e in the manuscript.

For the strengthening of E_{max}/E_0 near the M-Pt patch, we observed a significant increase with areal fraction Θ_c. This is mainly due to the point-sink effect as predicted by Equation S3. As shown in Figures S2b and S2c, the E_{max}/E_0 values typically locates near the edges and corners of the M-Pt plating. This increase of E_{max}/E_0 will significantly enhance the local reaction rate there, which also matches

our experimental observation. On the contrary, when Θ_c is large, the condition of simulated system resembling the point-sink potential solution failed, and we cannot find significant E_{max} enhancement in the simulation domain, as suggested by the theory we stated. In summary, Equation S3 suggests that, the smaller the M-Pt area, the higher the E_{max}/E_0 enhancement will be, and the E_{max} typically locate on the edges and corners of M-Pt.

Next, we assess the areal reaction efficiency of the M-Pt geometry. Although the E_{max} can be increased by using a smaller M-Pt area, the area available for reaction will be reduced. Therefore, there is a trade-off between E_{max} and M-Pt area, and we expect to see a maximum average E-field by varying the M-Pt electrode area. This effect can be assessed by considering E_{ave}/E_0, where E_{ave} is the average E-field value over the whole bottom electrode area. By plotting the trend of E_{ave}/E_0 as in Figure 6e in manuscript, we find that E_{ave}/E_0 reaches a peak when the Θ_c is in range from 0.1 to 0.3. This maximum exists because of the trade-off between small reaction area versus high induced E-field values. If the design wants both reaction area and induced E-field to be high, we will land at a maximum areal average E-field value on the bottom electrode. This experimental observation is in accordance with this simulation prediction.

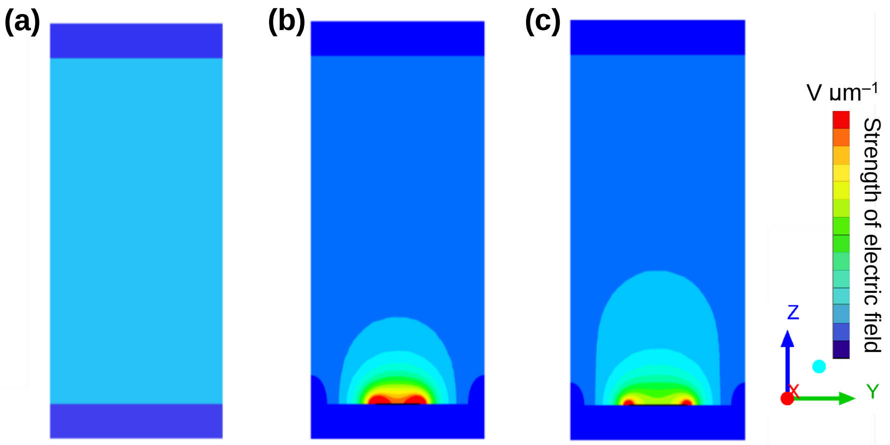

Figure S2. The E-field distributions in electrolyte on electrode surface with model without bubble of (a) U-Pt case, (b) M-Pt case (L/D = 0.25), and (c) M-Pt case (L/D = 0.3333). The uniform light blue

color in the U-Pt case is the E_0 value, which is the reference E-field strength. We can see the significant enhancement of E-field from the M-Pt construction.

Results of Case 2. E-field distribution of U-Pt vs M-Pt with bubble

The second scenario simulated a hemispheric bubble on the U-Pt and M-Pt patches, respectively, i.e., using model (b). The detailed E-field distributions in the domain are then compared. We study the case where the H_2 gas bubble is of size 100um, which does not completely block the 125um M-Pt plating, as shown in Figure S3.

As expected from the potential theory, the bubble has enhanced the E-field near the locations surrounding the bottom electrode, around the equator of the bubble near the Pt plating. In both U-Pt case (Figure S3a) and M-Pt case (Figure S3b), we observed a strengthening of the local E-field near the bubble, but the enhancement is less pronounced in the U-Pt case (Figure 3Sa). The M-Pt case enhancement is much stronger, creating a stronger E-field (*i.e.*, red color) around the M-Pt plate. This strong enhancement is because of the bubble directly modified the Θ_c condition, which the value is much smaller due to the partial blockage of M-Pt by the bubble. This creates a condition better resemble the point-sink solution Equation S3, allowing the solution to remain valid at even smaller displacement vector **r**. This condition is true even when the **r** is as small as the little exposed M-Pt patches, providing an environment of very large E-field enhancement at very small **r**. Therefore, we see a stronger strengthen effect due to the aforementioned potential theory on a point-sink.

In summary, with this simulation case, we explained how the point-sink theory help us to understand a small blocking bubble can create a better Θ_c condition on the M-Pt patch, where this

much smaller value promotes a better E-field enhancement. Nevertheless, since the bubble is blocking reaction area, its existence on the M-Pt impacts the reaction area size.

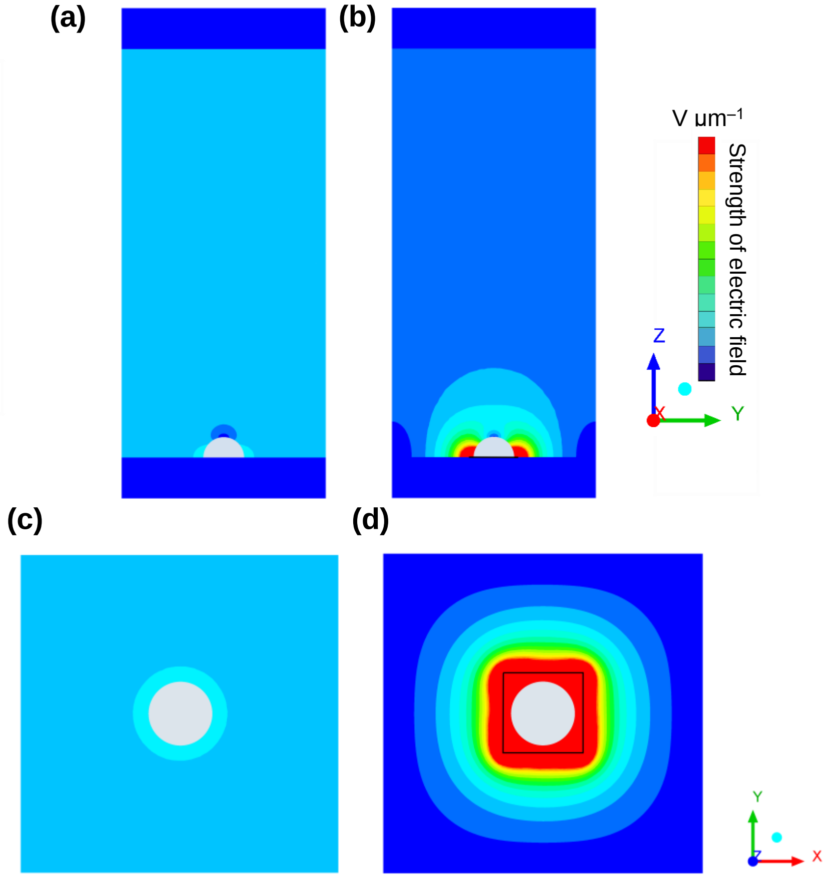

Figure S3. The E-field distributions in the system with a bubble. A hemispheric bubble of diameter 100 μm is situated at the center of the bottom electrode (a) U-Pt case and (b) M-Pt case. The cross-sectional plane here is the Z-Y plane passing through the center of the bubble. The top and bottom blue regions are the electrodes. (c) and (d) are the respective cases on the X-Y plane right on the bottom electrodes. In the U-Pt cases (a) and (c), the equatorial location of the bubble induces a stronger E-field according to potential theory expectation. For the M-Pt cases (b) and (d), there is further enhancement of the E-field due to the point-sink condition created by the M-Pt patch and the bubble.

3. Materials and samples characterization

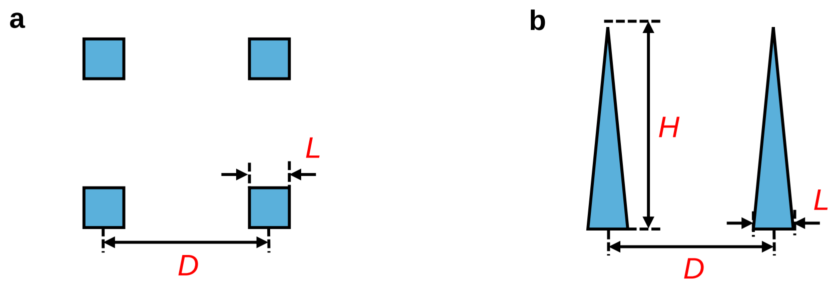

Samples	Shape of catalyst islands	Occupation of catalyst (Θ_c)	L (μm)	H (μm)	D (μm)
M1-Pt	square	0.25	125	N/A	250
M2-Pt	square	0.25	5	N/A	10
M3-Pt	square	0.1736	125	N/A	300
M4-Pt	square	0.1111	125	N/A	375
M5-Pt	square	0.0625	125	N/A	500
M6-Pt	square	0.015625	125	N/A	1000
Mt-Pt	triangular	0.15	300	6000	740
M-Ru	square	0.25	125	N/A	500
Mt-Ru	triangular	0.15	300	6000	740
Mt-PtS	triangular	0.15	300	6000	740

Table S2. Detailed geometrical parameters of all the catalysts studied in this work. Schematics showing key parameters for mosaic catalysts composed of (a) square catalyst regions and (b) triangular catalyst regions. For the catalysts made of square regions, the occupation of catalyst (Θ_c) is defined as area of catalyst divided by area of the whole surface, *i.e.*, $\Theta_c = L^2/D^2$. N/A stands for not available.

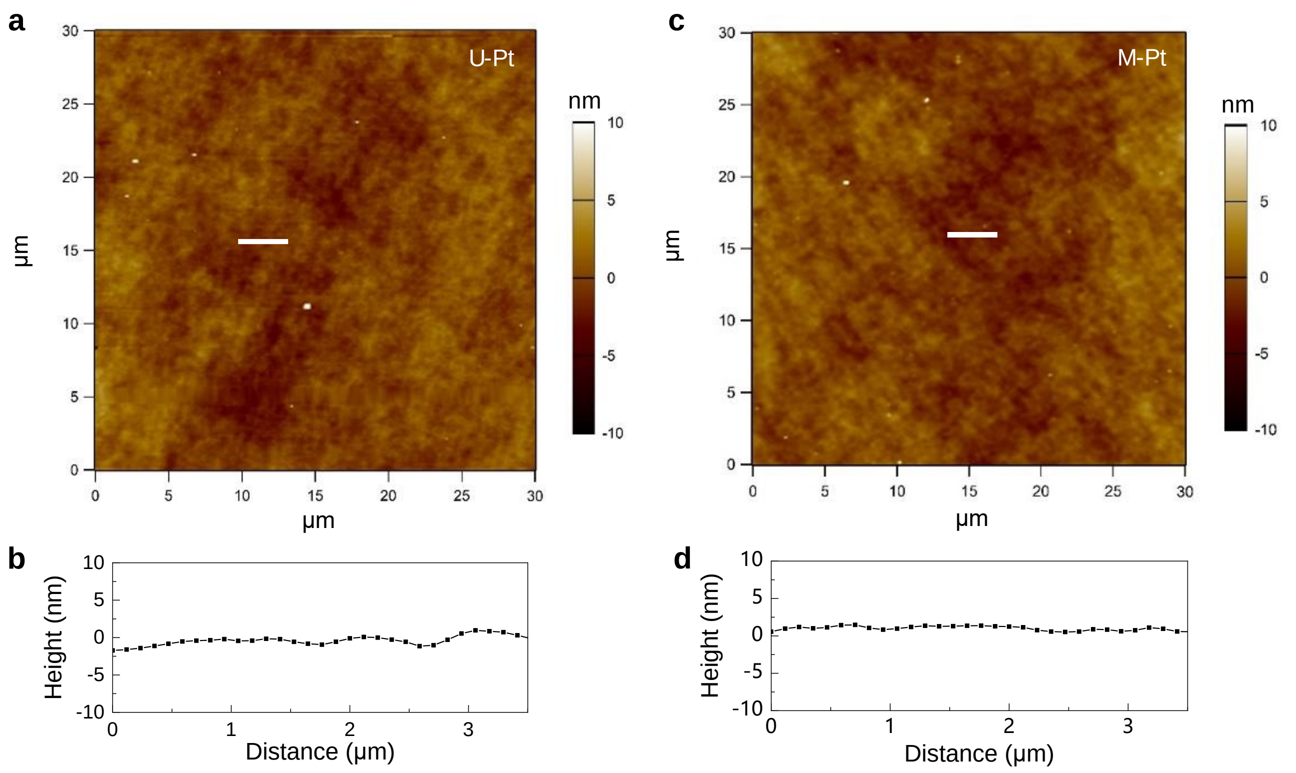

Figure S4. AFM characterization of Pt catalysts. AFM images of (a) U-Pt and (c) M-Pt, as well as height profiles of (b) U-Pt and (d) M-Pt. The RMS roughness values are 1.228 nm and 1.216 nm for U-Pt and M-Pt, respectively. The results show that the morphologies of different Pt samples are similar.

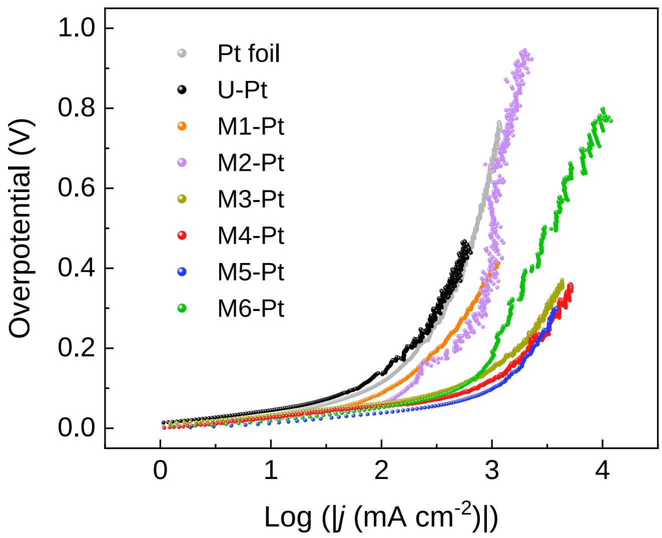

Figure S5. Tafel curves of different Pt catalysts, including Pt foil, U-Pt, and mosaic Pt catalysts with different geometrical parameters of spatial structure.

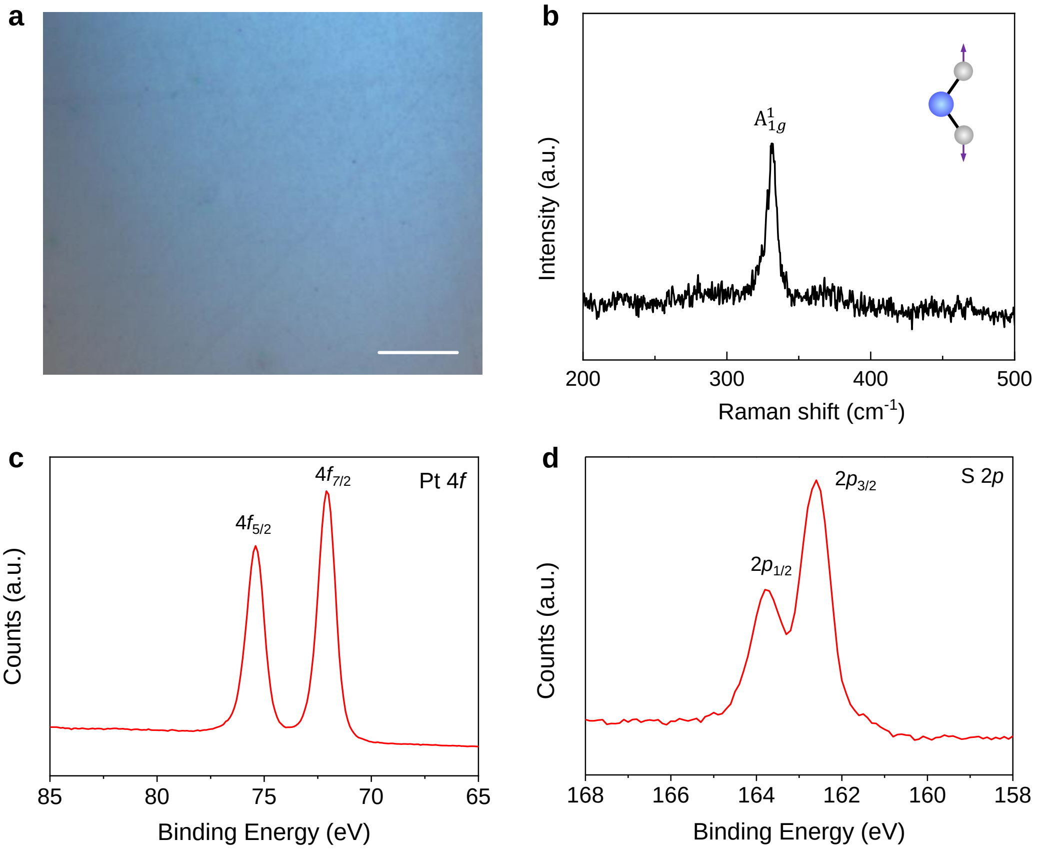

Figure S6. Characterization of CVD grown PtS. (a) An OM image. (b) Raman spectrum. The peak at 331.7 cm^{-1} is attributed to the A_{1g}^1 mode of PtS. Laser wavelength is 532 nm in Raman experiments. (c, d) XPS spectra of (c) Pt 4f and (d) S 2p. The scale bar in a is 10 μm.

4. Electrochemical tests

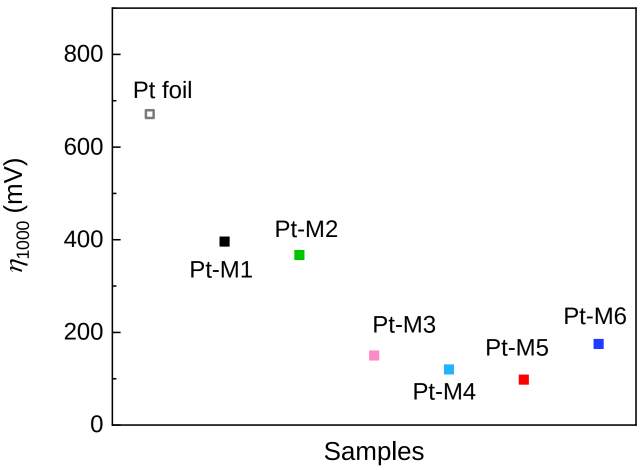

Figure S7. A summary of the η_{1000} values (defined as overpotentials at j_{geo} = 1000 mA cm^{-2}) of different Pt catalysts for HER in a 0.5 M H_2SO_4 solution.

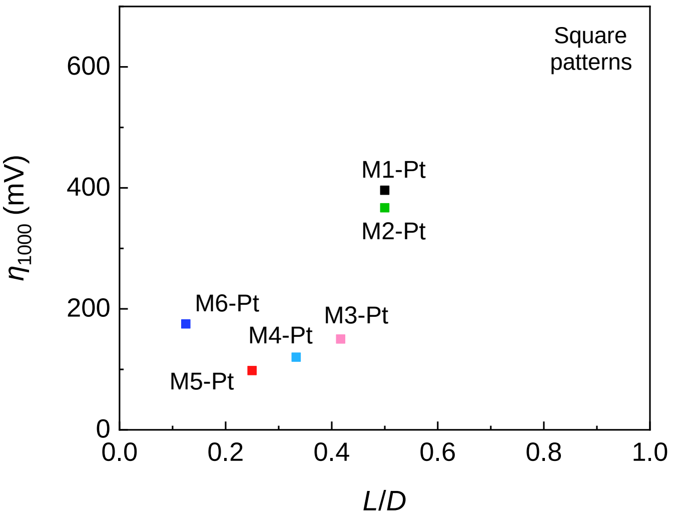

Figure S8. A summary of η_{1000} values of Pt catalysts and their *L*/*D* ratios**.** The results show that η_{1000} shows a correlation with *L*/*D*. Note that L^2/D^2 equals Θ_c in the case of mosaic Pt catalysts. They do not show unidirectional correlations because both the *L*/*D* ratios as well as the mass transfer abilities influence the catalytic performance of mosaic Pt catalysts.

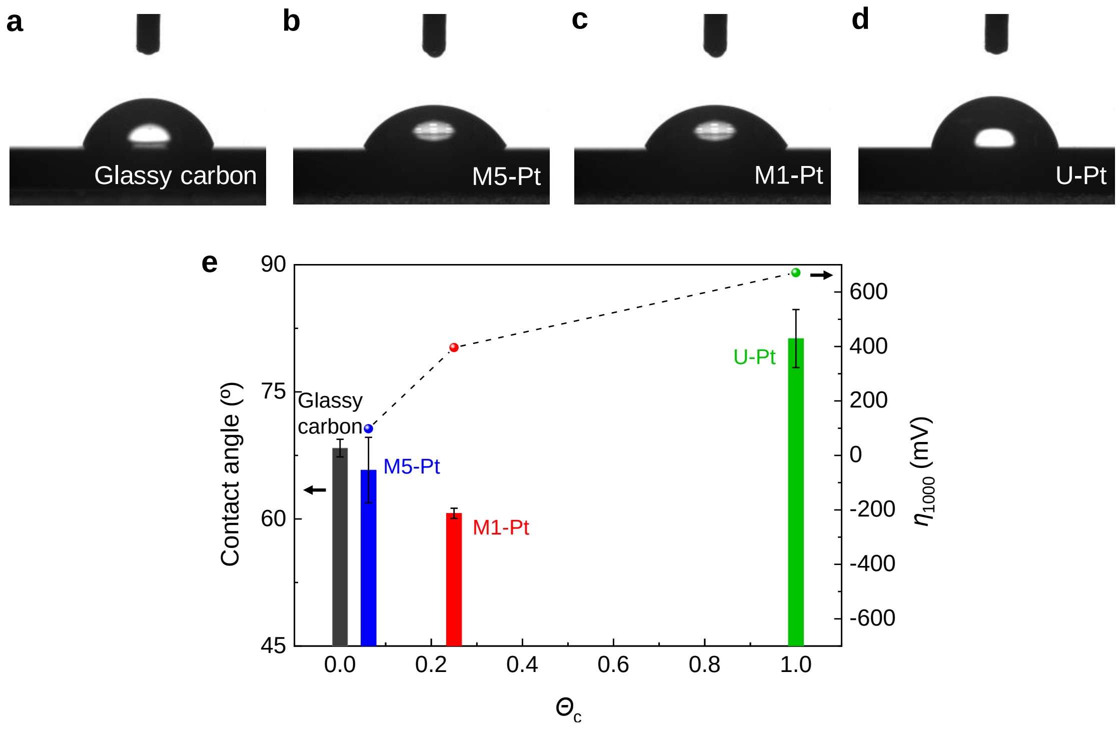

Figure S10. Pt catalysts with different Θ_c and contact angles (CAs), where CA is a key factor influencing mass transfer ability of catalysts. (a-d) Optical images of a water droplet (4 μL) on different sample surfaces. (e) A summary of Θ_c and CA values of different samples. The M1-Pt shows the smallest CA for water droplet. However, M5-Pt has the best catalytic performance, suggesting mass transfer is not the only factor for determining the catalytic performance of these catalysts.

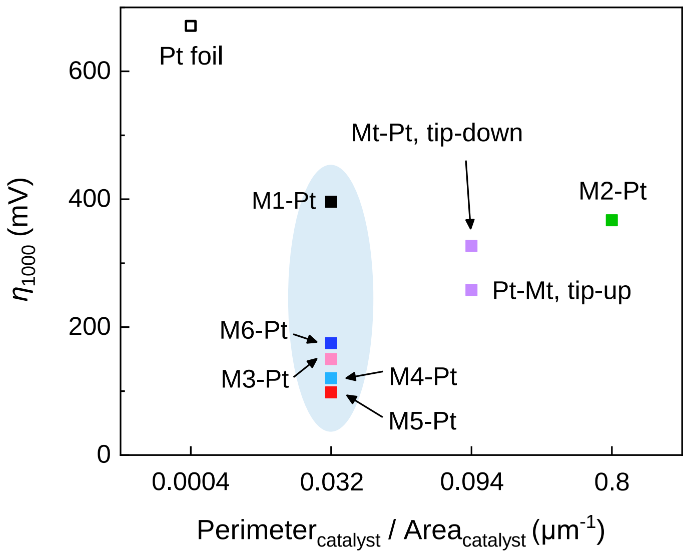

Figure S11. A summary of the η_{1000} values of different Pt samples plotted against their Perimeter$_{catalyst}$/Area$_{catalyst}$ values (defined as ratio of catalyst perimeter to catalyst area). The results show that there is no correlation between η_{1000} and Perimeter$_{catalyst}$/Area$_{catalyst}$. Note that, although M1-Pt, M3-Pt, M4-Pt, M5-Pt, and M6-Pt have the same Perimeter$_{catalyst}$/Area$_{catalyst}$, their η_{1000} are different, suggesting that Perimeter$_{catalyst}$/Area$_{catalyst}$ is not a key factor determining performance of these catalysts.

Table S3 | A comparison of the specific activity of M1-Pt catalyst at an overpotential of 50 mV obtained in this work compared with the 20 wt% Pt/C film catalysts reported in the literature. These data were plotted in Figure 2e.

Catalyst	Specific activity (mA cm^{-2})	Ref.
**M1-Pt**	**16.7**	**This work**
**U-Pt**	**1.4**	**This work**
Pt/C	1.3	[10]
Pt/C	0.8	[11]
Pt/C	10.6	[12]
Pt/C	0.7	[13]
Pt/C	11.6	[14]
Pt/C	8.1	[15]

5. Transferability of hydrogen bubbles and the effects

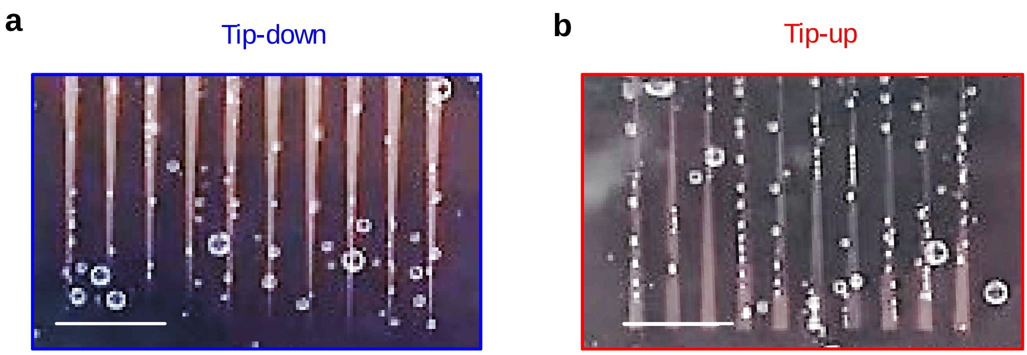

Figure S11. Photos showing H_2 bubbles on Mt-Pt with opposite orientations at a j_{geo} of 10 mA cm^{-2}. (a) Tip-down Mt-Pt. (b) Tip-up Mt-Pt. The tip orientations of Mt-Pt are referred to the flow direction of H_2 bubbles. These two samples have the same morphology and chemical composition, making the placed orientation the only different parameter between them. Tip-down and tip-up samples show similar H_2 bubbles with small sizes at low current densities. Both scale bars are 2 mm.

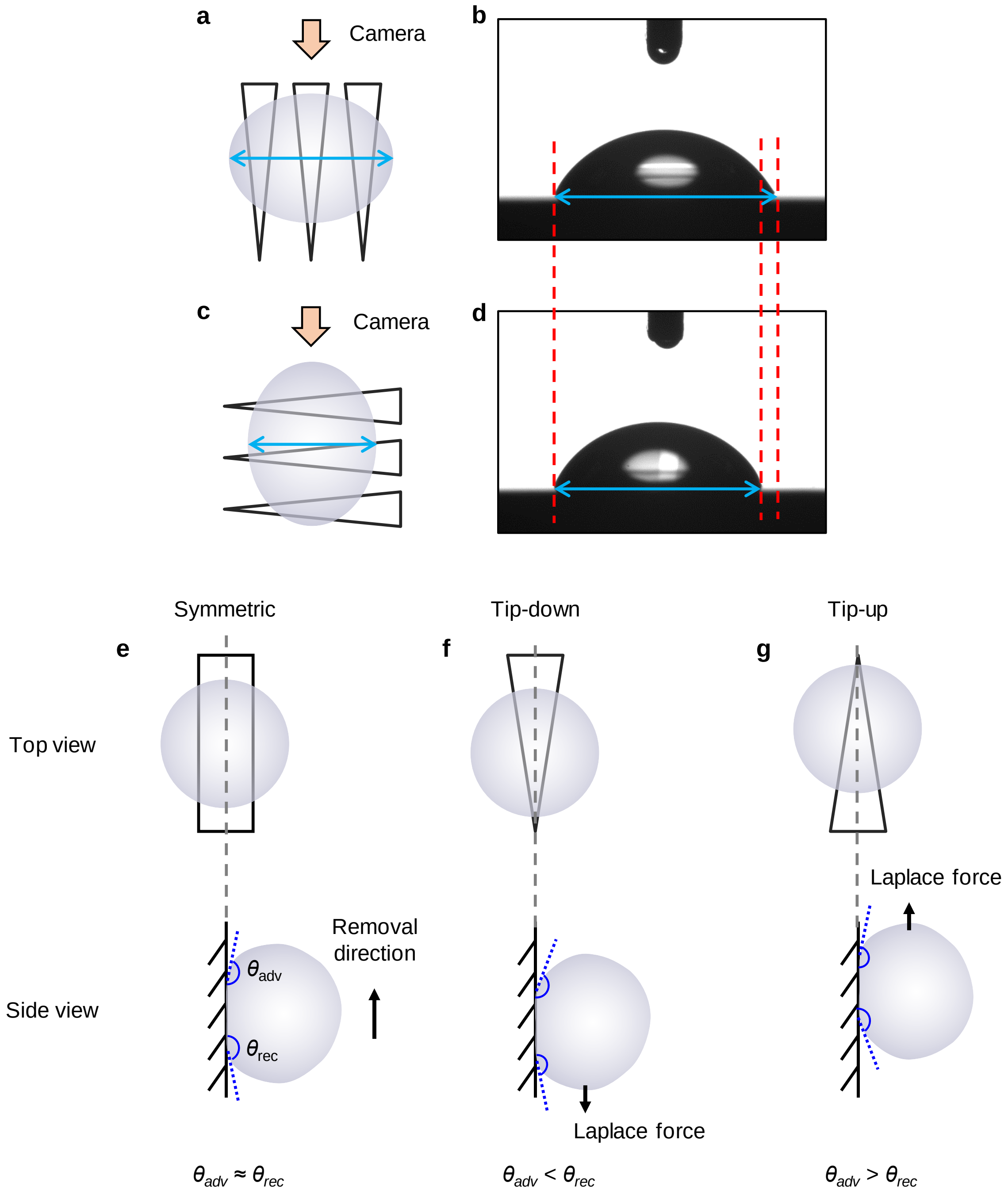

Figure S12. Explanation of different transfer ability of H_2 bubbles on tip-down and tip-up Mt-Pt samples. (a-d) Water droplets on the Mt-Pt surface are deformed, from observation directions (a, b) parallel to the axis of triangular islands and (c, d) perpendicular to the axis of triangular catalyst islands. (e-g) Such a deformation indicates that a negative Laplace force is exerted on H_2 bubbles on tip-down Mt-Pt while a positive Laplace force is exerted on tip-up Mt-Pt, leading to different mass transfer abilities of the two Mt-Pt catalysts in the HER.

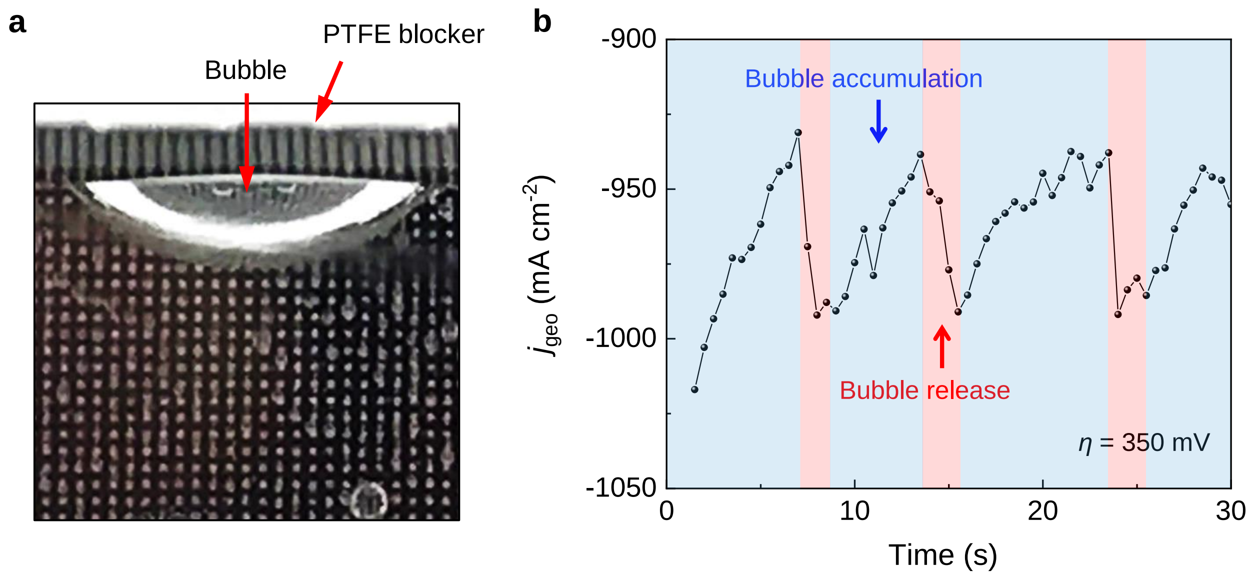

Figure S13. Relationship between HER performance and the accumulation/release of H_2 bubbles on M1-Pt. (a) A optical microscopy image shows a big bubble accumulation adhering to the surface of M1-Pt, which is caused by an aerophilic PTFE blocker. (b) *i-t* curve shows that HER performance of M1-Pt is tuned by accumulation/release of the big bubbles. The area of catalyst covered or blocked by H_2 bubbles is ~8%, delivering a ~7% decrease in current density.

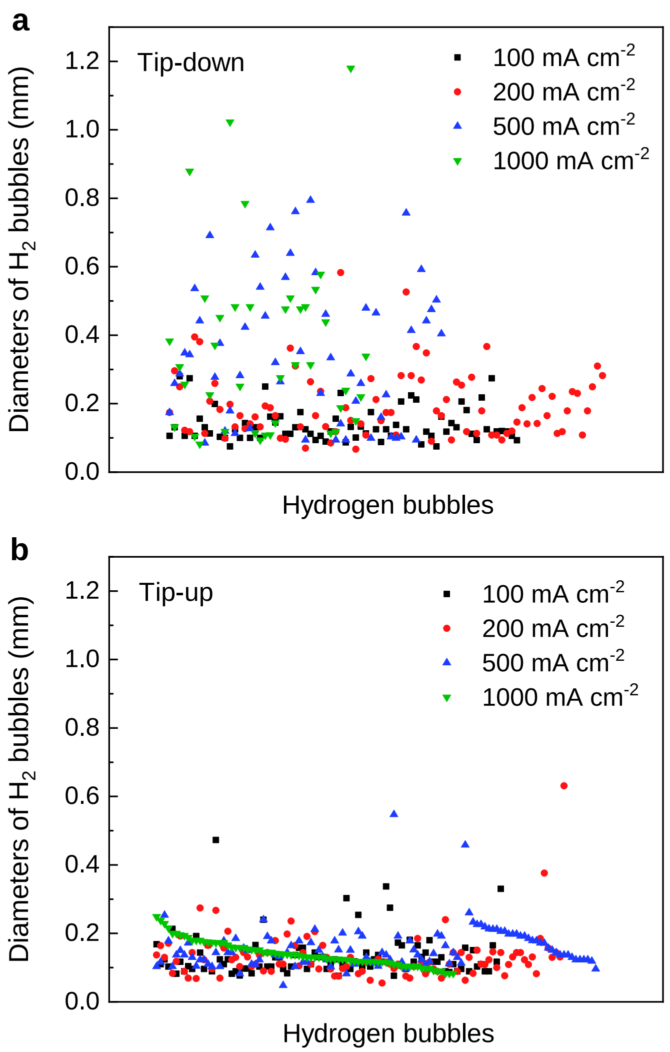

Figure S14. Diameters of H_2 bubbles on (a) tip-down and (b) tip-up Mt-Pt samples at different current densities for calculating the average radius and r_H^2 in Figure 5d. The results show that the tip-up Mt-Pt shows smaller H_2 bubbles compared to the tip-down Mt-Pt. For each case, more than 40 bubbles were statistically analyzed.

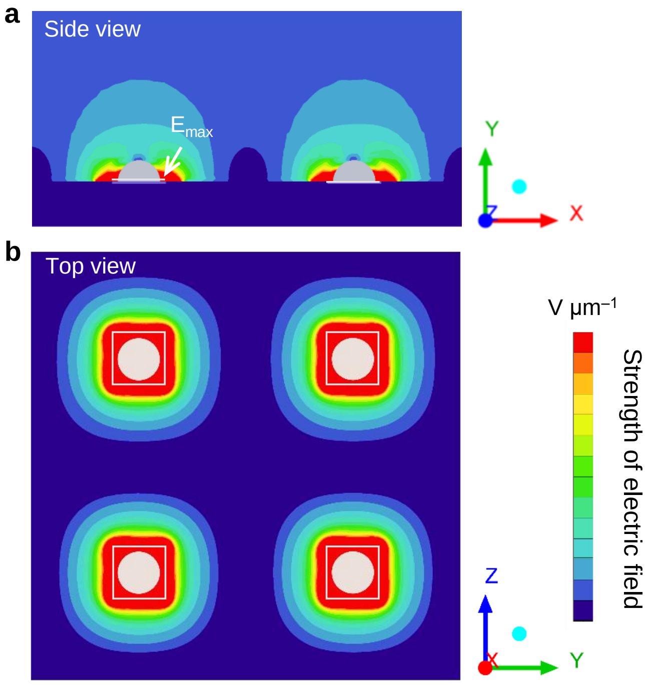

Figure S15. Simulations of the electric field distribution on the M-Pt with hemispheric bubbles sitting on, where the regions in electrolyte is displayed. (a, b) Electric field distribution of M-Pt arrays from the (a) side view and (b) top view, where a hemispheric bubble with a diameter of 100 μm is situated on the top of each square Pt region. The top view shows the cross-sectional plane passing through the center of the bubble, in the electrolyte regions. The results show that the maximum electric field is further enhanced due to the existence of bubbles. The Pt regions are shown by white lines in b.

Movie S1. Comparison of the movement of H_2 bubbles on the surfaces of U-Pt and M1-Pt catalysts.

Movie S2. Comparison of the movement of hydrogen bubbles on the surfaces of Mt-Pt (*i.e.*, triangular shape catalysts) with tip-up and tip-down orientations.

6. Catalytic performance normalized by electrode surface areas

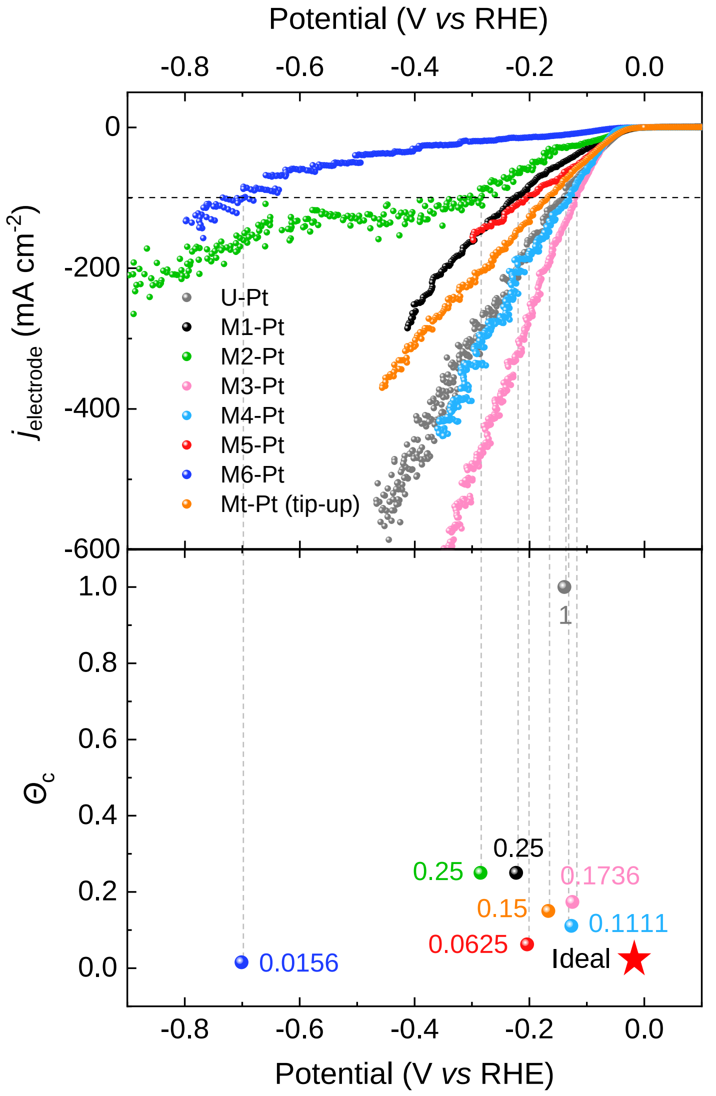

Figure S16. HER performance of U-Pt and M-Pt with different Θ_c values (defined as occupation ratio of catalysts on a support). (a) Polarization curves of different Pt catalysts. (b) A summary of Θ_c and overpotentials at $j_{electrode}$ = 100 mA cm^{-2}. The $j_{electrode}$ is current density determined by projected surface area of electrode, which is a performance index for practical use.

7. Supporting References

[1] L. Tang, T. Li, Y. Luo, S. Feng, Z. Cai, H. Zhang, B. Liu, H.-M. Cheng, *ACS Nano* **2020**, *14*, 4646.

[2] W. Liu, D. Lin, A. Pei, Y. Cui, *J. Am. Chem. Soc.* **2016**, *138*, 15443.

[3] P. Zou, Y. Wang, S. W. Chiang, X. Wang, F. Kang, C. Yang, *Nat. Commun.* **2018**, *9*, 464.

[4] F. Che, J. T. Gray, S. Ha, N. Kruse, S. L. Scott, J.-S. McEwen, *ACS Catal.* **2018**, *8*, 5153.

[5] M. Rosso, T. Gobron, C. Brissot, J. N. Chazalviel, S. Lascaud, *J. Power Sources* **2001**, *97-98*, 804.

[6] J. Newman, K. E. Thomas-Alyea, *Electrochemical Systems, 3rd Edition*, John Wiley & Sons, Inc., **2004**.

[7] S. Kim, W. J. Dong, S. Gim, W. Sohn, J. Y. Park, C. J. Yoo, H. W. Jang, J.-L. Lee, *Nano Energy* **2017**, *39*, 44.

[8] J. D. Jackson, *Classical electrodynamics*, John Wiley & Sons, **1998**.

[9] L. D. Landau, E. M. Lifshitz, *Electrodynamics of continuous media*, Pergamon Press, **1984**.

[10] Z. Li, J.-Y. Fu, Y. Feng, C.-K. Dong, H. Liu, X.-W. Du, *Nat. Catal.* **2019**.

[11] L. Xiu, W. Pei, S. Zhou, Z. Wang, P. Yang, J. Zhao, J. Qiu, *Adv. Funct. Mater.* **2020**, 1910028.

[12] D. Liu, X. Li, S. Chen, H. Yan, C. Wang, C. Wu, Y. A. Haleem, S. Duan, J. Lu, B. Ge, P. M. Ajayan, Y. Luo, J. Jiang, L. Song, *Nat. Energy* **2019**, *4*, 512.

[13] D. H. Kweon, M. S. Okyay, S. J. Kim, J. P. Jeon, H. J. Noh, N. Park, J. Mahmood, J. B. Baek, *Nat. Commun.* **2020**, *11*, 1278.

[14] A. Alinezhad, L. Gloag, T. M. Benedetti, S. Cheong, R. F. Webster, M. Roelsgaard, B. B. Iversen, W. Schuhmann, J. J. Gooding, R. D. Tilley, *J. Am. Chem. Soc.* **2019**, *141*, 16202.

[15] Z. Zhang, G. Liu, X. Cui, B. Chen, Y. Zhu, Y. Gong, F. Saleem, S. Xi, Y. Du, A. Borgna, Z. Lai,

Q. Zhang, B. Li, Y. Zong, Y. Han, L. Gu, H. Zhang, *Adv. Mater.* **2018**, *30*, e1801741.
